# Traitement antalgique des crises vaso-occlusives à domicile avant l’arrivée en consultation

**DOI:** 10.11604/pamj.2020.37.127.25062

**Published:** 2020-10-05

**Authors:** Alima Yanda Anastasie Nicole, Eposse Ekoube Charlotte, Ngatsing Christelle Shanti, Mandeng Ma Linwa Edgar, Françoise Ngnedjou Nwabufor Foute, Ngou Patrick, Mbono Ritha, Djike Puepi Yolande, Epee Patricia, Wete Estelle, Koki Ndombo Paul Olivier

**Affiliations:** 1 Institut Supérieur de Technologie Médicale, Yaoundé, Cameroun,; 2 Centre Mère et Enfant Fondation Chantal BIYA, Yaoundé, Cameroun,; 3 Centre de Prise en Charge des Drépanocytaires, Hôpital Laquintinie, Douala, Cameroun,; 4 Faculté de Médecine et des Sciences Pharmaceutiques, Université de Douala, Douala, Cameroun,; 5 Faculty of Health Sciences, University of Buea, Buea, Cameroun,; 6 Université Adventiste Cosendai, Nanga Eboko, Cameroun,; 7 Faculté de Médecine et des Sciences Biomédicales, Yaoundé, Cameroun

**Keywords:** Traitement, antalgique, crises vaso-occlusives, Treatment, analgesic, vaso-occlusive crises

## Abstract

**Introduction:**

les crises vaso-occlusives (CVO) constituent le principal motif de consultation et d´hospitalisation chez les enfants drépanocytaires et nécessite le plus souvent une prise en charge à domicile avant l´arrivée en consultation. Le but de cette étude était de décrire l´attitude thérapeutique à domicile des enfants drépanocytaires reçus en consultation pour des CVO.

**Méthodes:**

il s´agissait d´une étude transversale descriptive et analytique, qui s´est déroulée aux urgences pédiatriques du Centre Mère et Enfant de la Fondation Chantal Biya sur une période de 4 mois allant de février à mai 2018. Nous avons procédé à un échantillonnage consécutif. Tous les patients drépanocytaires venus consulter pour CVO ont étés inclus.

**Résultats:**

cent cinquante-deux patients ont été enrôlés dans l'étude. La tranche d´âge la plus représentée était celle de 5-10 ans. Quatre-vingt-deux patients (54%) arrivaient plus de 24h après le début des crises et 70 (46%) dans les 24 premières heures; 80% (n=122) de notre échantillon avaient eu un traitement antalgique à domicile. Nous avons retrouvé une escalade thérapeutique dans 31,2% de cas. Le recours aux médicaments de la prescription médicale (75,4%, n=92) prédominait au 1^er^ recours. La posologie des antalgiques n´était pas correcte dans 67% de cas (surdosage dans 70% des cas). Seul 33% des antalgiques utilisés à domicile étaient administrés à de bonnes posologies.

**Conclusion:**

l´étude démontre que la prise en charge des CVOs par les patients à domicile est inadéquate. Des mesures doivent être prises pour garantir que tout patient drépanocytaire puisse gérer efficacement les CVOs mineures à modérées à domicile.

## Abstract (en)

**Introduction:**

vaso-occlusive crises (VOCs) are the primary reason for admission and hospitalization in children with sickle cell disease and most often they require home care before arriving to consultation. The purpose of this study was to describe home-based therapy for children with sickle cell disease admitted with VOCs.

**Methods:**

we conducted a descriptive and analytical cross-sectional study, in the pediatric emergencies at the Mother and Child Centre of the Chantal Biya Foundation (CBF) over a 4-month period from February to May 2018. Consecutive sampling was used. All patients with sickle cell disease presenting with VOCs were included in the study.

**Results:**

one hundred and fifty-two patients were enrolled. The most represented age group was 5- 10 years. Eighty-two patients (54%) presented more than 24 hours after the onset of the crises and 70 (46%) in the first 24 hours; 80% (n=122) of our sample had received analgesic treatment at home. Escalation of therapy was found in 31.2% of cases. The use of prescription medications (75.4%, n=92) was dominant as the 1^st^ recourse. The dosage for analgesics was not correct in 67% of cases (overdose in 70% of cases). The correct dosage of home-based analgesics was administered in only 33% of patients.

**Conclusion:**

this study shows that home-based management of VOCs is inadequate. Measures must be taken to ensure that all patients with sickle cell disease can effectively manage minor to moderate VOCs at home.

## Introduction

La drépanocytose, encore appelée anémie à hématies falciformes, est une affection héréditaire, transmise selon un mode autosomique récessif et survenant sur le chromosome 11 [1,2], caractérisée par une anémie hémolytique chronique liée à la présence dans le sang d´une hémoglobine anormale (HbS) en lieu et place de l´hémoglobine normale (HbA). C´est la maladie génétique la plus répandue dans le monde avec une nette prédominance au sein de la population noire [1,2]. La drépanocytose est fréquente au Cameroun mais sa prévalence exacte n´est pas connue. Un travail pilote de dépistage néonatal de la drépanocytose réalisé sur 40000 échantillons montre une incidence de 15,1% de porteurs du trait drépanocytaire et 0,7% naissances vivantes (en cours de publications). Les crises vaso-occlusives constituent une des complications aiguës les plus fréquentes de cette hémoglobinopathie. C´est le principal motif de consultation et d´hospitalisation des enfants drépanocytaires. Au Cameroun, une étude hospitalière réalisée par Mbassi *et al*. en 2015 dans 3 centres hospitaliers de Yaoundé montre une prévalence hospitalière de 52,19% de toutes les hospitalisations des drépanocytaires [3]. En effet, les CVO sont imprévisibles, répétées, parfois sévères, insoutenables et durables; ce qui fait de la drépanocytose une affection douloureuse et invalidante [4-6]. Elles constituent également une urgence thérapeutique qui nécessite une prise en charge rapide et bien codifiée [6]. Malgré un bon suivi des patients, le respect des règles hygiéno-diététiques et des protocoles thérapeutiques, les crises vaso-occlusives demeurent imprévisibles et leur évolution incertaine, nécessitant le plus souvent une gestion antalgique à domicile avant l´arrivée en consultation lorsque celles-ci persistent. Nous nous sommes intéressés à l´attitude thérapeutique à domicile des enfants drépanocytaires reçus en consultation pour des crises vaso-occlusives.

## Méthodes

**Population, lieu et durée d´étude:** nous avons réalisé une étude transversale descriptive et analytique sur une période de 4 mois allant de février à mai 2018. Elle s´est déroulée aux urgences pédiatriques du Centre Mère et Enfant de la Fondation Chantal BIYA à Yaoundé-Cameroun qui est un hôpital de référence pédiatrique situé à Yaoundé avec une unité de prise en charge de la drépanocytose qui reçoit 3000 drépanocytaires en suivi ambulatoire et 500-600 hospitalisations par an.

**Critères de sélection et échantillonnage:** nous avons procédé à un échantillonnage consécutif. Ont été inclus tous les enfants drépanocytaires reçus en consultation pour des CVO.

**Procédure et outils de collecte des données:** pour la collecte des données, nous avons utilisé une fiche technique individuelle préalablement établie. Ainsi que des guides pratiques des médicaments (Dorosz 2017; Vidal: édition 2014) pour la détermination des DCI des médicaments utilisés et l´évaluation de la qualité de la posologie des antalgiques administrés à domicile. Les variables d´intérêt étaient: le délai écoulé entre le début des CVO et l´arrivée en consultation, la fréquence du traitement antalgique des crises à domicile, les modalités du traitement antalgique à domicile: le nombre de recours thérapeutique, le type de recours thérapeutique, les antalgiques administrés à domicile, la posologie de ces antalgiques. Nous avons aussi recherché le niveau de scolarisation des parents/tuteurs, leur appartenance à une association des parents d´enfants drépanocytaires; le suivi médical correct des enfants.

**Définitions opérationnelles**

**Suivi régulier:** avoir fait au moins trois (03) consultations en dehors de toute urgence médicale au courant de l´année 2017.

**Recours thérapeutique:** approche thérapeutique relative à ce qui est fait pour palier à l´épisode morbide.

**Type de recours thérapeutique:** prescription médicale: elle fait référence au recours aux médicaments prescrits par le médecin traitant; automédication moderne: fait référence à l´utilisation des médicaments modernes par le parent lui-même sans avoir au préalable eu une prescription médicale; médecine traditionnelle/médecine alternative.

**Qualité de la posologie des antalgique:** bonne posologie: correspond au bon dosage et à la bonne fréquence par prise des médicaments; mauvaise posologie: correspond au non-respect des modalités d´administration de l´antalgique (dosage par prise et la fréquence de prises).

**Gestion et analyse des données:** les données recueillies étaient saisies dans le logiciel CS Pro 7.0; puis importées vers le logiciel SPSS version 23.0 pour analyse. Les tests de Chi carré et de Fisher étaient utilisés pour les mesures d´association entre les variables catégorielles. Le seuil de significativité était fixé à 5% et l´IC était égale à 95%.

**Considérations éthiques:** une autorisation d´étude a étȅ accordée par les services administratifs du CME/FCB. Les parents/tuteurs des enfants éligibles ont clairement étȅ informés de l´objet de notre étude et leur consentement obtenus en vue de leur inclusion dans notre étude. Les informations recueillies ont étȅ traitées avec la plus grande confidentialitȅ et ne seront utilisées que dans un but scientifique.

## Résultats

**Figure 1:**
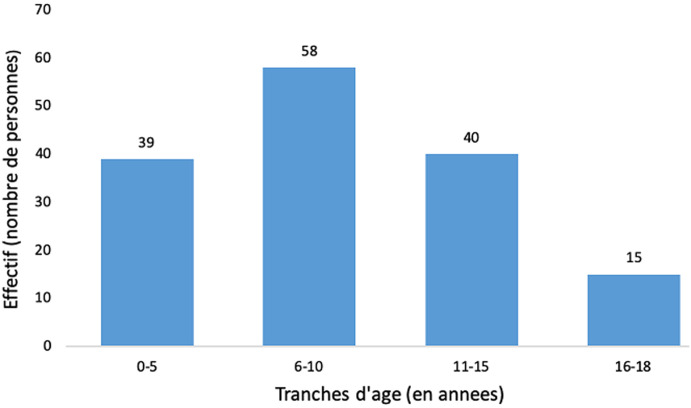
répartition des patients en fonction des tranches d´âges

**Caractéristiques sociodémographiques:** notre population d´étude était composée de 152 enfants dont 69 garçons et 83 filles soit un sex-ratio de 0,83. La tranche d´âge la plus représentée était celle de 5-10 ans (40,1%) (Figure 1). L´âge moyen était de 9,14±4,27 ans; avec des extrêmes allant de 1 an à 18 ans. Les patients étaient originaires de la région du centre dans 54,6% de cas.

**Délai de consultation:** cinquante-quatre pour cent (n=82) des patients arrivaient plus de 24h après le début des CVO et 46% (n=70) dans les premières 24 heures. Le temps moyen écoulé entre le début des crises et l´arrivée en consultation était de 38,6h; avec une médiane de 27,5h et des extrêmes allant de 2h à 168h (=7 jours).

**Prévalence du traitement antalgique des CVO à domicile:** cent vingt-deux patients avaient eu recours à un traitement antalgique à domicile avant l´arrivée en consultation, soit une prévalence du traitement antalgique à domicile estimé à 80%. Les antalgiques les plus utilisés à domicile étaient les AINS (49,7%) avec en tête de file le diclofénac (27,7%), suivi de l´ibuprofène (16,7%).

**Modalités du traitement antalgique à domicile:** au cours des CVOs, 68,8% (n=84) des patients ayant eu recours à un traitement antalgique à domicile avaient pris un antalgique avant l´arrivée en consultation. Par contre nous retrouvions une escalade thérapeutique dans 31,2% de cas: respectivement 24,6% (n=30) et 6,6% (n=8) étaient à leur 2^e^ et 3^e^ recours thérapeutiques. Le nombre moyen de recours thérapeutiques était de 1,38. Pour ce qui est du type de recours thérapeutiques: au 1^er^ recours thérapeutique, 75,4% (n=92) de patients prenaient des antalgiques issus d´une prescription médicale. Tandis que 23,8% (n=29) faisaient une automédication moderne; au 2^e^ recours thérapeutique, 63,1% (n=24) réalisaient une automédication moderne tandis que la prescription médicale et le recours aux médicaments traditionnels représentaient respectivement 26,3% (n=10) et 10,6% (n=4); au 3^e^ recours, les types de recours thérapeutiques étaient repartis comme suit: 50%(n=4) automédication moderne, 37,5% (n=4) prescription médicale et 12,5% (n=1) la médecine traditionnelle.

Les antalgiques principalement utilisés au cours de la prise en charge des CVO étaient les AINS (49,7%) suivi du paracétamol seul (24,5%); l´association du paracétamol avec l´ibuprofène ou la codéine représentait respectivement 5,8% et 8,4%. Nous avons également noté l´utilisation en automédication des médicaments autres que les antalgiques pour la PEC des CVO. Il s´agissait du métronidazole, de l´amoxicilline, de la nystatine et de l´hydroxycarbamide. Les antalgiques étaient mal administrés (posologies incorrectes) dans 67% de cas (n=102) et administrés au bon dosage par prise et à la bonne fréquence de prises dans seulement 33% de cas. Les antalgiques employés aux mauvaises posologies étaient administrés dans 70% de cas à des doses élevées et 30% en sous dosage. Ainsi nous avons retrouvé en analyse multivariée que les antalgiques étaient administrés aux bonnes posologies d´une part quand ils venaient d´une prescription médicale (p-value=0,002) et d´autres part quand ils étaient administrés par des parents d´enfants drépanocytaires suivis (p-value=0,007) (Tableau 1).

**Tableau 1:** facteurs associés à la bonne administration des antalgiques à domicile: analyse multivariée

Variables	Qualité de la posologie		Odds ration ajusté (IC=95%)	P-value ajusté
	**Mauvaise**	**Bonne**		
Suivi médical	30	38	0,23(0,08-0,67)	0,007S
**Type de recours médicamenteux**				
1. Automédication moderne	26	1	14,76(1,65-131,52)	0,010S
2. Prescription médicale	45	42	0,12(0,03-0,45)	0,002S
**Niveau scolaire des parents/tuteurs**				
1. Secondaire	40	16	3,21(0,72-14,30)	0,120NS
2. Supérieur	23	24	1,94(0,42-8,85)	0,390NS
Appartenance à une association des parents d'enfants drépanocytaires	9	12	0,83(0,28 -2,40)	0,730NS

IC: intervalle de confiance, S: statistiquemment significatif, NS: non-significatif

## Discussion

Au terme de notre travail, 152 patients ont fait partie de l´étude; 54,6% était de sexe féminin soit un sexe ratio de 0,83. Mbassi Awa *et al*. [3] avaient retrouvé une prédominance masculine. Les résultats peuvent être différents d´une série à une autre car la drépanocytose est une maladie héréditaire, à transmission autosomique récessive co-dominante, qui se fait indépendamment du sexe. La tranche d´âge la plus représentée était celle de 5 à 10 ans, pour un âge moyen de 9,14 ans±4,2. Ces résultats se rapprochent de ceux retrouvés par Mbassi Awa *et al*. et Nacoulma *et al.* [3,7] où la tranche d´âge de 5 à 10 ans était prépondérante, avec une moyenne de 8±4 ans. Cela pourrait s´expliquer par le fait que les crises vaso occlusives sont fréquentes chez les enfants [8]. Cinquante-quatre pourcent des patients (n=82) arrivaient plus de 24 heures après le début des crises tandis que 46% (n=70) dans les 24 premières heures. Le délai moyen était de 38,6h, une médiane de 27,5h; avec des extrêmes allant de 2h à 168h (7 jours). Le délai entre le début des crises et l´arrivée en consultation pourrait avoir été influencé par l´intensité de la douleur de l´enfant, l´apparition d´autres signes (asthénie, les difficultés respiratoires etc.) et les possibilités financières. Ce délai moyen montre que les parents essayent en général d´observer l´évolution des douleurs en essayant un traitement, sauf si elles sont d´emblée sévères. Cent vingt-deux patients avaient bénéficié d´un traitement antalgique à domicile avant l´arrivée à l´hôpital, soit une prévalence de 80%. Ce résultat se rapproche de celui de Shapiro *et al*. [9] en 1995 qui a retrouvé qu´en dépit de la facilité d´accès aux soins de la population américaine, 89% des crises douloureuses étaient traitées à domicile par les patients eux-mêmes.

Les résultats obtenus par Dampier *et al*. [10] en 2002 au cours d´une étude concernant les enfants et adolescents drépanocytaires qui tenaient un calendrier de douleur à domicile sont également concordants aux nôtres: 60 à 90% de leurs CVO n´étaient pas gérés à l´hôpital. Ce résultat peut être expliqué par le fait que les patients drépanocytaires bénéficient d´une éducation thérapeutique de la maladie qui inclue la gestion des douleurs à domicile et une prescription d´antidouleurs. De plus du fait de multiples hospitalisations, ils s´accommodent à l´usage de ces antidouleurs qu´ils manipulent à domicile en cas de douleur. L´utilisation d´un antalgique était retrouvée dans 68,8% (n=84) de cas au cours de la prise en charge à domicile. Ce résultat était inférieur à celui de Dampier *et al*. [10]. Dans son travail, la prise d´un antalgique à domicile était la stratégie la plus utilisée par 85% des patients. Par ailleurs, 24,6% (n=30) et 6,6% (n=8) avait respectivement eu recours à un 2^e^ et à un 3^e^ médicament avant l´arrivée à l´hôpital. Le nombre moyen de recours thérapeutique étaient de 1,38. Ce résultat se rapproche de celui de Commeyras *et al*. [11] en 2006; qui avait trouvé que le nombre moyen de recours dans la population générale camerounaise était de 1,4. Nous avons retrouvé une escalade thérapeutique dans 31,2% de cas faite de la prescription médicale, de l´automédication moderne et de la pharmacopée traditionnelle.

Ainsi 75,4% (n=92) des patients avaient recours en 1^ère^ intention aux médicaments issus d´une prescription médicale et 23,8% à l´automédication. En 2^e^ intention, la tendance s´inversait; 63,2% (n=24) des patients pratiquaient l´automédication et 26,3% (n=10) avaient recours à la prescription médicale. La première approche était donc le recours à la prescription médicale et la 2^e^ approche; l´automédication. Ces résultats sont différents de ceux retrouvés par Ngou au cours d´une étude menée en 2011 sur l´itinéraire de recours aux soins des patients reçus au service des urgences du CME-FCB [12]. Dans cette dernière, lors d'un épisode morbide, 49,6% des patients avaient recours en premier intention à l'automédication; la raison évoquée dans 67% de cas était l´habitude au recours [12]. Cette différence pourrait s´expliquer par le fait que le travail de Ngou ne traitait pas de pathologies chroniques. Les antalgiques les plus utilisés à domicile étaient les AINS (49,7%) avec en tête de file le diclofénac (27,7%), suivi de l´ibuprofène (16,7%). Le paracétamol était également, fréquemment utilisé seul (24,6%) ou en association avec la codéine (8,3%) ou l´ibuprofène (5,8%). Ces résultats se rapprochent de ceux de Dampier *et al*. [10]. Cette étude révèle que les molécules les plus consommées étaient le paracétamol, la codéine et l´ibuprofène de façon isolée ou en association.

Gbadoé *et al*. [13] au Togo ont également trouvé que les moyens thérapeutiques les plus fréquemment utilisés étaient les salicylés (61,8%), le paracétamol (37%), les autres AINS (15,1%). L´utilisation fréquente des AINS dans notre étude reflète probablement la prescription habituelle des praticiens dans la prise en charge ambulatoire des CVO qui peut varier d´un pays à l´autre. La proportion de patients ayant eu recours aux médicaments traditionnels (médecine alternative) était faible (0,8%, 10,5% et 12,5% au premier, deuxième et troisième recours respectivement), probablement due au fait de l´éducation thérapeutique des parents concernant les complications éventuelles des traitements traditionnels. La qualité de la posologie était mauvaise dans la grande majorité de cas; 67% des antalgiques utilisés à domicile étaient mal administrés (posologies incorrectes), parmi lesquels 70% était donné en surdosage et 30% en sous dosage. Seulement 33% des antalgiques étaient administrés à un bon dosage et une bonne fréquence de prises. Ainsi nous avons retrouvé que le suivi médical et le recours aux médicaments prescrits étaient les facteurs associés à la bonne administration des antalgiques à domicile. L´administration des antalgiques aux mauvaises posologies était le fait du recours à l´automédication moderne.

## Conclusion

La prise en charge antalgique à domicile des crises vaso-occlusives par les sujets/parents de drépanocytaires est fréquente. L´usage des AINS et du paracétamol est prépondérante, mais le plus souvent à de mauvaises posologies. Un accent particulier doit être mis sur l´éducation thérapeutiques des parents/sujets drépanocytaires.

### Etat des connaissances sur le sujet

- Les CVOs affectent la fréquentation scolaire et peuvent conduire aux absences à l'école (21% des jours d'école). Parmi les absences liées à la douleur, les deux tiers se sont produits lorsque la douleur a été prise en charge à domicile;
- Le traitement des CVOs à domicile se fait à plus de 70% avec l´usage des analgésiques. Les techniques les plus fréquemment utilisées pour la gestion de la douleur comprenaient les salicylates (61,8%), le paracétamol (37%), les anti-inflammatoires non stéroïdiens (15,1%), les vasodilatateurs et la pentoxifylline (5,4%);
- En Afrique sub-Saharienne et en particulier au Cameroun, ou la prévalence de la drépanocytose est importante et les barrières d´accès aux soins qui sont souvent d´ordre financiers sont présent, peu d´études ont évalué les attitudes thérapeutiques des patients souffrant de CVOs avant l´arrivée à l´hôpital (un pool qui comprends pourtant la plus grande partie des épisodes de CVOs).

### Contribution de notre étude à la connaissance

- Les AINS étaient principalement utilisés dans près de la moitié des patients. Les posologies des antalgiques administrés dans plus de la moitié des cas n´étaient pas correctes et étaient pour la plupart des surdosages;
- Les facteurs associés à la bonne administration des antalgiques étaient le suivi médical et la prescription médicale;
- L´étude démontre que la prise en charge des CVOs par les patients à domicile est inadéquate. Des mesures devraient être prises pour que tout patient drépanocytaire puisse être en mesure de gérer les crises mineures a modérées efficacement à domicile, par exemple des séances d´éducation thérapeutiques sur la prise en charge des CVO à domicile.

## Remerciements

Notre profonde gratitude va aux participants pour leur contribution.
